# Dissolved Cellulose Couples with Flow and Capillarity to Affect Ionic Liquid Crystallization: An X‑ray Diffraction and Raman Spectroscopy Study

**DOI:** 10.1021/acs.jpcb.5c08715

**Published:** 2026-05-19

**Authors:** Adriana Šturcová, Nikolay Kotov, Vladimír Raus, Alexander Zhigunov

**Affiliations:** Institute of Macromolecular Chemistry, 86879 Czech Academy of Sciences, Heyrovského nám. 2, Prague 162 00, Czech Republic

## Abstract

Cellulose is a semicrystalline polymer with domains controlled by a cooperative hydrogen-bond network and by solvophobic forces that do not energetically favor solubility. Ionic liquids dissolve such domains in a nonderivatizing way, and many ionic liquids also meet the criteria of green solvents. The crystallization behavior of 1-butyl-3-methylimidazolium chloride (bmimCl) was investigated in the presence of cellulose and added water. It was hypothesized correctly that the structural transitions in bmimCl during low-temperature treatment would reflect the interactions with either solute. Mixtures with cellulose at concentrations of 1 or 3 wt % and with added water concentrations of either 0.0, 0.6, or 2.7 wt % were subjected to two low-temperature treatments, i.e., repeated exposure at either −25 °C or −17 °C with room temperature in between. Such mixtures were investigated by Fourier-transform or dispersive Raman spectroscopy and wide-angle X-ray scattering. The two different treatments induced fluid flow with either more laminar or more turbulent character. The more laminar flow led to the extended AA butyl chain conformation in bmimCl and to the orthorhombic crystal structure O; the more turbulent flow led to the GA conformation and either the metastable stressed monoclinic M or the stable monoclinic M0 crystal structure. The presence of cellulose can shift the butyl chain conformation toward the more extended one; such a shift might be an effect of interactions, or cellulose macromolecules might be acting indirectly. By containing the mixtures in two types of glass vessels with different internal diameters (2 mm or 9 mm), the coupling of the fluid flow to capillarity phenomena was confirmed. Such findings are important for ionic liquid phase transitions, for cellulose–ionic liquid interactions, and for structuring of materials by flow, thus attaining better control of material properties across length scales, starting at nanodimensions through to micro- and macrodimensions.

## Graphical abstract

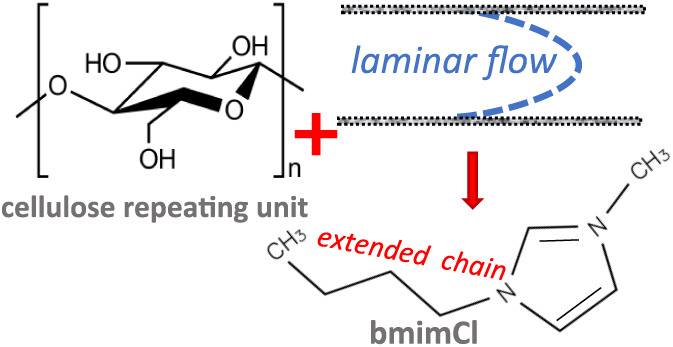

## Introduction

Cellulose is an example of a semicrystalline polymer that is renewable and serves as a resource for valuable materials and chemicals.^1^ Before cellulosic biomass can be exploited, it has to be dissolved and often also depolymerized, but it is challenging to do so since its solubility in common solvents is limited.^2^ In polymer dissolution, just like for low-molecular solid compounds, the free energy of the solution has to be lower than the free energy of the two separate phases, and the process is driven by entropy.^3^ It is also useful, however, to consider the balance of intermolecular interactions—breaking those interactions that are within cellulose structures and forming new intermolecular interactions between cellulose and its solvent.^2,3^ In addition, not only thermodynamic control but also kinetic control of the dissolution process can often be very important for macromolecules;^4,5^ this situation is very different from that of low-molecular-weight compounds since such compounds diffuse and mix more easily. As a result, polymers do not dissolve instantaneously, and when they do dissolve, it is a multistep process that involves mass-transport events.^5^

In order to optimize the dissolution process for the intended applications of cellulose, it is important to fully understand its dissolution mechanism. For a long time, it was argued that breaking the strong and cooperative hydrogen bonds within and between cellulose macromolecules was the most important part of the process.^6^ About 15 years ago, Lindman and his coworkers^2,7–10^ reviewed the physicochemical aspects of polymer dissolution and concluded that not only hydrogen-bonding interactions are relevant but hydrophobic interactions also play an important part, since cellulose can be considered amphiphilic. In the equatorial direction of its glucopyranose ring, cellulose has a hydrophilic character due to the hydroxyl groups, while in the axial direction of the ring, cellulose has a hydrophobic character due to the axial positions of the hydrogen atoms of C–H bonds. It is then not surprising that in the structure of one of the polymorphs of cellulose–cellulose I, the macromolecular chains interact via intermolecular hydrogen bonds within one plane, while they are stacked via hydrophobic forces between the C–H groups in the direction perpendicular to that plane.^11–14^ A suitable solvent needs to break both types of interactions; therefore, ionic liquids are good solvents for cellulose^6^ since they are amphiphilic, like cellulose,^15^ and they are predicted to use both the cation and the anion to penetrate and disassemble the cellulose structure without derivatizing it.^16^

Continued effort has been made to prove that the hydrogen-bonding network in cellulose is disturbed or significantly altered upon dissolution, and it was met with success.^17^ More recently, proof of hydrophobic interactions between cellulose and its solvent has also been sought.^11,18–25^ For imidazolium-containing ionic liquids, the search for hydrophobic interaction means the search for interaction between the cellulose d-glucose ring and the aromatic ring of the imidazolium cation. Such interactions were often assumed in the literature, but until recently, they were typically documented only by theoretical simulations, and experimental evidence was scarce.^11,18–24^ However, in the year 2020, Kotov et al.^25^ reported the collapse of signals in solid-state nuclear magnetic resonance (ssNMR) spectra from carbon atoms of the imidazolium ring in the ionic liquid 1-butyl-3-methylimidazolium chloride (bmimCl) that was mixed with cellulose. These signals were originally split in the ssNMR spectra of neat bmimCl, and further to that, small differences in the chemical shift were detected. Such changes were interpreted as an indication of the cellulose–bmimCl interactions that alter the geometry of the crystallized bmimCl. The carbons of the butyl chain in bmimCl were not affected to the same extent, which led to the conclusion that it is the carbohydrate–aromatic stacking between the aromatic imidazolium rings of the ionic liquid cations and the d-glucose units of cellulose that takes place, while the role of the aliphatic chain could not be specified in more detail at that point. Subsequent analysis of the values of relaxation times obtained for the cellulose/bmimCl mixture by the same authors^25^ indicated that the dynamics of all bmimCl carbons slowed down upon the addition of cellulose. Such a change in dynamics had been previously observed for other mixtures of cellulose with ionic liquids and is usually considered to be an impediment in cellulose dissolution, as mentioned above.^26,27^ We can hypothesize that the retardation in motion of all carbons, including those in the butyl chain, might be a consequence of bmim^+^–cellulose interactions, or alternatively, the aliphatic chain may play a more active role. Interactions similar to carbohydrate–aromatic stacking reported by Kotov et al.^25^ had been proposed for other polysaccharides prior to that report.^28^

Other aspects of cellulose solutions can be investigated in addition to the dissolution mechanism. When cellulose is dissolved and processed, the different phases of the process, such as homogenization, transport, and formation, require flow, while the flow significantly affects the performance of the resultant material because it affects its structuring already at the nanoscale.^29^ Further to that, the addition of a polymer to a fluid can be used to influence the flow of that fluid.^4^ Reynolds showed in 1883 ^30^ that, as fluid velocity increases, its trajectories change from a laminar flow to a turbulent one. If the setup of the flow is the same, then the transition from the steady linear pattern of the laminar flow to the dynamic and sinuous pattern of the turbulent flow depends solely on one parameter that is nondimensional and became known as the Reynolds number, *Re*:

1 $$Re=\frac{\rho Ul}{\eta }$$

where *ρ* and *η* are the fluid density and viscosity, respectively, and *U* and *l* are the characteristic velocity and length scales of the flow, respectively. The transition from laminar to turbulent flow is accompanied by abrupt changes in the characteristics of the flow, especially by the rise in the friction factor.^31^ Thus, the friction loss in the turbulent regime is much higher than in the laminar regime, and techniques for the reduction of this turbulent drag are of great importance in practical applications.

Polymers have long been reported as efficient agents for turbulent drag reduction. Various uses of polymers within the field of flow dynamics can be found—ranging from applications where polymers are used to optimize transport in the oil industry or aviation^32^ to those where instabilities are explored to understand the mixing of fluids in microfluidic systems.^31^ Even though research on drag reduction has a long history, there are important advances that have been made relatively recently, and there are issues that still need to be addressed, e.g., clarifying the applicability of viscous vs elastic mechanisms in polymer drag reduction.^30^ Where fluid dynamics is concerned, linear long-chain macromolecules with flexible backbones are known to be the most effective turbulent drag-reducing molecules, and the drag reduction behavior of rigid polymers such as cellulose is less explored; however, some rigid macromolecules have also been observed to cause drag reduction.^31^

In addition to their ability to dissolve cellulose, ionic liquids are fluids that have very interesting phase behavior.^33–35^ Since they are complex fluids—they are not built from single neutral molecules but from two types of interacting ions^36^ —they usually have complex crystallization behavior with transitions between different polymorphs^33,34,37,38^ that can depend on their temperature history and also on the presence of meso- or nanoconfinement.^39,40^ It is necessary to understand such behavior so that the use of ionic liquids in applications can be optimized; therefore, the current study might be relevant to the flow phenomena that are present whenever cellulose is processed by solution casting or spinning. Further to this, Wang et al.^41^ successfully exploited the reversible crystallization of an ionic liquid, bmimCl, in the development of a tough-stiff switchable ionogel where cellulose acted as a regulator that promoted the formation of ionic liquid crystals. In addition, control of ionic liquid crystallization has an important role in, e.g., electrochemical energy storage applications, gas detection, and CO_2_ fixation.^40^

By studying structural transitions and other physical phenomena both in the neat ionic liquid bmimCl and in its mixtures with cellulose or with cellulose and added water, the study presented here aimed: (1) to advance the understanding of the factors affecting cellulose dissolution and of the phenomena and processes leading to it; (2) to gain more information on the impact of flow on structuring in mixtures of cellulose with ionic liquid, and on the impact a rigid polymer can have on the flow of a fluid, in the search for answers to some important questions within the field of polymer dynamics and the field of flow turbulence; (3) to improve the knowledge of dynamics, structure, and phase-transition behavior of ionic liquids themselves. An attempt was made to fulfill these aims by applying the techniques of Raman spectroscopy and wide-angle X-ray diffraction (WAXS).

## Materials and Methods

### Materials

1-Butyl-3-methylimidazolium chloride (bmimCl, Fluka, Sigma-Aldrich, ≥99.0% (HPLC); Scheme 1) and water (LC–MS CHROMASOLV, Fluka, Sigma-Aldrich) were kept at 4 °C prior to sample preparation and used as received. Cellulose Avicel PH-101 (Fluka Biochemika, Sigma-Aldrich; Scheme 1) was dried at 80 °C in a vacuum for 24 h prior to dissolution.

**Scheme 1:**
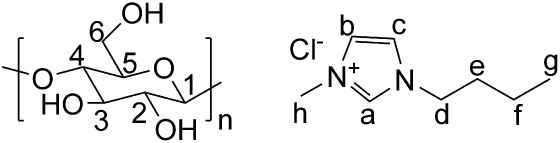
Chemical Structure of Anhydroglucose (AGU) Unit of Cellulose (Left) and of bmimCl (Right)

### Sample Preparation

Into a preweighed vial containing a magnetic stirring bar, a certain amount of melted (at ca. 90 °C) bmimCl was placed via a syringe, and the net bmimCl weight was determined. Into the intensively stirred bmimCl, heated to ca. 90 °C, the calculated amount of Avicel PH-101 was slowly added. Then, the mixture was stirred for about 45 min to achieve complete cellulose dissolution. Note that the vial was argon-flushed before and after the addition of the individual components. Where relevant, calculated amounts of water were added via a microsyringe, and the exact amount of water was again determined by weighing. The mixtures with water were mixed at ca. 80 °C. From the vial, samples of the mixtures were transferred using a syringe into 2 mm capillaries, 9 mm vials, or both. The sample labeling and composition are presented in Table 1.

**Table 1:** Content of Cellulose and Water in the Samples Used

Sample	Weight fraction of cellulose (%)	Molar fraction of cellulose^a^ (%)	Weight fraction of added water (%)	Molar fraction of added water (%)
**C1**	1.0	1.1	0.0	0.0
**C3**	3.0	3.2	0.0	0.0
**C3–W1**	3.0	3.1	0.6	5.4
**C3–W3**	2.9	2.5	2.7	21.3

a Calculated as the molar fraction of anhydroglucose units in the final mixture.

### Low-Temperature Treatments

The samples C1, C3, C3–W1, and C3–W3 (letter C here stands for cellulose and letter W stands for water) in vials and in capillaries were first kept at room temperature and then either at the temperature of −17 °C or −25 °C. Two low-temperature treatments were used, each consisting of nine steps (Table 2). In between the steps, WAXS experiments, FT-Raman, and dispersive Raman spectroscopy were performed. An attempt was made to achieve the same temperature history for all the samples and all of the three characterization methods. However, the times needed to record a satisfactory Raman spectrum and WAXS diffractogram could be very different. For this reason, we had to slightly adjust the conditions of the low-temperature treatment for each method, choosing step sequences that would make correlation between all three methods possible. Please note that, in previous work by this group of authors,^39^ neat bmimCl was treated as follows: after the sample preparation, the vial and capillary were cooled to laboratory temperature and kept at this temperature for 16 h (step 1); then at −25 °C for 16 h (step 2); then for an additional 56 h (step 3); then for an extra 7 days (step 4); and finally for 2 weeks (step 5). In this work, we will refer to such treated samples of bmimCl, which will be compared with binary and ternary mixtures of this ionic liquid with cellulose and added water.

**Table 2:** A Description of Low-Temperature Treatments Used

Step	Treatment 1	Treatment 2
1	room temperature, 16 h	room temperature, 16 h
2	–25 °C, 16 h	–25 °C, 16 h
3	–25 °C, 3 days	–25 °C, 3 days
4	–25 °C, 7 days	–25 °C, 6 days
5	–25 °C, 7 days	–17 °C, 3 days
6	–25 °C, 23 days	–17 °C, 3 days
7	–25 °C, 22 days	variable temperature, 2 days
8	–25 °C, 4 days	–25 °C, 24 days
9	–25 °C, 6 days	–25 °C, 7/10 days

### FT-Raman Spectroscopy

A Raman module attached to the Thermo Nicolet 6700 FTIR (Fourier-transform infrared) spectrometer was used. The spectrometer had a silicon-coated CaF_2_ beamsplitter and was purged with dry air. The module (NXR FT-Raman) was equipped with an air-cooled InGaAs (indium–gallium-arsenide) detector, and an NIR (near-infrared) excitation laser with a wavelength of 1064 nm in the 180° scattering sample geometry was employed. The spectra were acquired on samples in glass containers (9 mm vials or 2 mm capillaries) with a resolution of 8 cm^–1^; a typical spectrum contained 256 scans.

### Dispersive Raman Spectroscopy

Ambient-pressure Raman spectra at various temperatures were obtained on a Renishaw In-via Raman microspectrometer with an Ar^+^ ion laser with an excitation line at 514.5 nm.

### WAXS

Wide-angle X-ray scattering (WAXS) experiments were performed using a pinhole camera (Molecular Metrology System, Rigaku, Japan) attached to a microfocused X-ray beam generator (Osmic MicroMax 002) operating at 45 kV and 0.66 mA (30 W). The camera was equipped with a removable and interchangeable imaging plate 23 × 25 cm (Fujifilm). The experimental setup covered the momentum transfer (*q*) range of 0.25–3.5 Å^–1^, where *q* = (4π/λ) sinθ, where λ = 1.54 Å is the wavelength, and 2θ is the scattering angle. Calibrations of the center and sample-to-detector distance were made using Si powder. Samples were measured in transmission mode.

## Results

FT-Raman spectroscopy was performed on samples in capillaries with an internal diameter of 2 mm and, where possible, also on samples in glass vials with a 9 mm internal diameter (as indicated in the text). WAXS measurements were performed on the capillaries only.

### Mixtures Undergoing Treatment 1

#### Mixtures of bmimCl with Cellulose

Mixture C1, with a composition of 1 wt % cellulose in bmimCl, was subjected to Treatment 1 both in a vial and in a capillary. Raman spectra corresponding to selected steps of Treatment 1 in the vial sample are displayed in Figure 1 (left), and for the capillary sample in Figure 1 (right). All of the displayed regions in the spectra of Figure 1 showed band narrowing as the temperature treatment progressed in time. In the right-hand panel of Figure 1, only the region between 900 and 400 cm^–1^ is displayed since primarily bands in this region were used for subsequent analysis. In neat bmimCl, the Raman bands at 730 cm^–1^ and 627 cm^–1^ correspond to the anti–anti (AA) conformation of the butyl group, while the bands at 701 cm^–1^, 605 cm^–1^, and 503 cm^–1^ are typical of the gauche–anti (GA) rotational isomer.^36,42^

**Figure 1:**
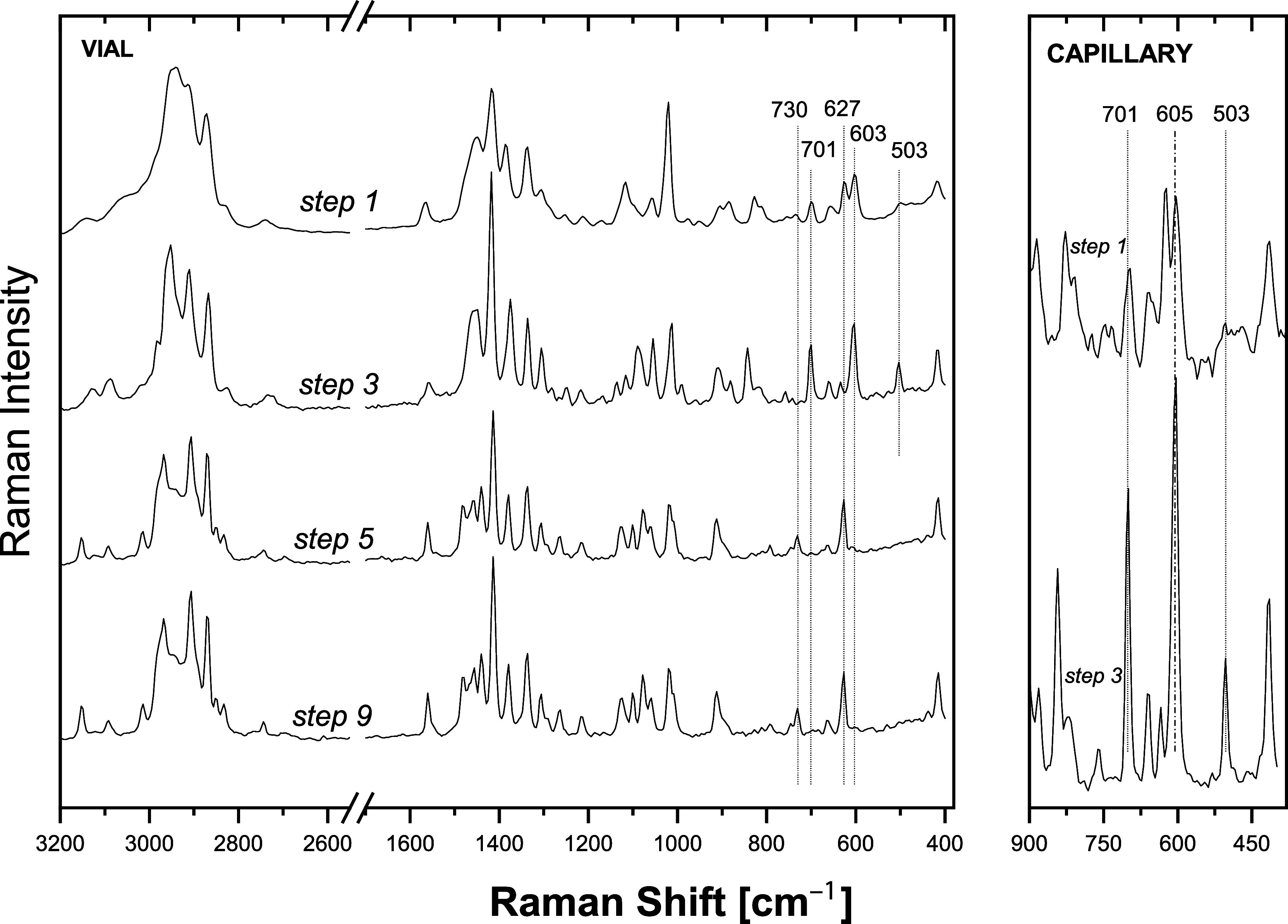
FT-Raman spectra of the 1 wt % cellulose/bmimCl mixture in a 9 mm vial (left) and in a 2 mm capillary (right) acquired at the selected steps of low-temperature Treatment 1. The spectra were scaled and offset for clarity.

Spectra of mixture C1, both in a vial and in a capillary after step 1, confirmed that the two butyl-chain conformers—GA and AA—were in equilibrium, indicating that bmimCl in the binary mixture was amorphous.^36^ In Table 3, after step 2 (−25 °C for 16 h), it is recorded, based on Raman spectra, that the capillary sample was still amorphous, while the vial sample was inhomogeneous, displaying amorphous regions as well as those with a dominant GA conformation. After subjecting mixture C1 to −25 °C for a further 3 days (step 3 in Figure 1; Table 3), the dominant GA conformation was detected in both the vial and the capillary, while the capillary sample was not monitored after this step due to breakage. In the vial, a further 7 days at low temperature (step 4) led to an inhomogeneous structure of the mixture—some regions converted from GA to AA conformers (Table 3). After a further 2 weeks at −25 °C (step 5 in Figure 1 and Table 3), only the AA rotational isomers were detected, and the vial sample remained in this form until the end of observation (step 9 in Figure 1 and Table 3). The behavior of capillary sample C3 is also shown in Table 3; it was described by Kotov et al. previously^25^ and it is used in this work for comparison and discussion.

**Table 3:** Conformation of the Butyl Group and Crystal Forms in bmimCl, Cellulose/bmimCl, and Cellulose/bmimCl/Water Mixtures Obtained After Low-Temperature Treatment 1 (Compilation of This and Previous Work)

	neat bmimCl^a^			C1 1.0 wt % cellulose		C3 3.0 wt % cellulose^b^		C3–W1 cellulose/bmimCl/water 3.0/96.4/0.6 wt %			C3–W3 cellulose/bmimCl/water 2.9/94.4/2.7 wt %	
	vial	capillary		vial	capillary	capillary		vial	capillary^b^		capillary^b^	
step	RS^c^	RS	WAXS	RS	RS	RS	WAXS	RS	RS	WAXS	RS	WAXS
1	am^d^	am	am	am	am	am		am	am		am	
2	AA		M0	am + GA	am	GA		GA + am	GA + am		am	
3	AA		M0	GA	GA	GA		GA	GA		am	
4	AA	GA	M0	AA + GA		GA		GA	GA+AA		am	
5			M0	AA		GA	M0	GA	AA		am	
6				AA		GA	M0	GA	AA	O	am	
7				AA		GA	M0	GA	AA	O	am	
8										O		am
9				AA				GA	AA + ph.s.			

a Kotov et al.^39^ Reproduced from ref 39. Copyright 2016 American Chemical Society.

b Kotov et al.^25^ Reproduced from ref 25. Copyright 2020 American Chemical Society..

c Raman spectroscopy.

d Amorphous.

#### Mixtures of bmimCl with Cellulose and Added Water

Mixture C3–W1 in a vial with a composition of 3.0/96.4/0.6 wt % of cellulose/bmimCl/water (Table 1) was amorphous after step 1 of Treatment 1. After step 2, domains with a dominant GA conformation started appearing, and the sample was fully converted to the GA form after step 3, remaining in this form until the end of observation (step 9). This behavior is documented in Figure 2 (left) and Table 3.

**Figure 2:**
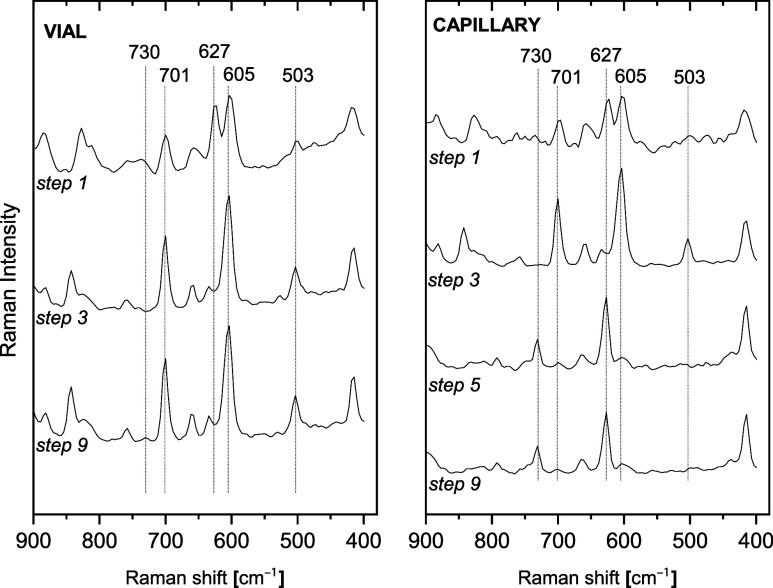
FT-Raman spectra of the cellulose/bmimCl/water mixture (3.0/96.4/0.6 wt %) in a 9 mm vial (left) and in a 2 mm capillary (right) acquired at the selected steps of low-temperature Treatment 1. The spectra were scaled and offset for clarity. Spectra 1, 3, and 5 in the right panel were adapted from ref 25, copyright 2020 American Chemical Society.

The same mixture, C3–W1, but placed in a capillary, was amorphous after step 1; amorphous domains and domains with the dominant GA conformer were formed after step 2; after step 3, all of the sample was in the GA conformation; after step 4, some of the GA domains converted to the AA conformation, and the AA conformation dominated after step 5 until the end of observation (step 9). The sample was found to be in orthorhombic crystal form (denoted as O in Table 3) after step 6 until the end of observation (step 9; see Figure 2 for Raman spectra and Table 3; diffractograms are not shown). Macroscopic phase separation into a liquid, colorless transparent phase and a solid, white-colored phase was observed 5 months after step 9, and dispersive Raman spectroscopy confirmed that the liquid phase was enriched in water while the solid phase contained bmimCl with the dominant AA conformation of the butyl chain (spectra not shown).

Mixture C3–W3 (containing 2.9/94.4/2.7 wt % of cellulose/bmimCl/water) in a capillary remained in the amorphous form throughout the low-temperature Treatment 1.

### Mixtures Undergoing Treatment 2

When mixture C1 (1 wt % of cellulose in bmimCl) in a capillary was subjected to a modified temperature treatment (low-temperature Treatment 2 in Table 2), the conformation after step 8 was found to be GA (Table 4; a spectrum is not shown), the same conformation reached by this sample when subjected to the first four steps of Treatment 1 (see above).

**Table 4:** Conformation of the Butyl Group and Crystal Forms in bmimCl, Cellulose/bmimCl, and in Cellulose/bmimCl/Water Mixtures Obtained After Low-Temperature Treatment 2

	neat bmimCl^a^		C1 1.0 wt % cellulose		C3 3.0 wt % cellulose		C3–W1 cellulose/bmimCl/water 3.0/96.4/0.6 wt %		C3–W3 cellulose/bmimCl/water 2.9/94.4/2.7 wt %	
	capillary		capillary		capillary		capillary		capillary	
step	RS	WAXS	RS	WAXS	RS	WAXS	RS	WAXS	RS	WAXS
1	am^b^	am								
2		M0								
3		M0								
4	GA	M0								
5		M0		M/M0		M/M0		M		M
8			GA		GA		GA			
9				M/M0		M/M0				M0

a Kotov et al.^39^ Reproduced from ref 39. Copyright 2016 American Chemical Society.

b Amorphous.

Capillary mixture C3, containing 3 wt % of cellulose, was also found to be in conformation GA after step 8 of the low-temperature treatment (Table 4; a spectrum is not shown) —the same as the conformation detected after step 7 of Treatment 1.

When the mixture C3–W1 in a capillary was subjected to low-temperature Treatment 2, as described in Table 2, the dominant GA conformation was detected after step 8 (Table 4; a spectrum is not shown). This sample was found in the stressed monoclinic crystal form M^39^ after step 5 of Treatment 2. This was in contrast to the AA conformation and orthorhombic crystal form O^39^ that were reached when low-temperature Treatment 1 was applied.

Sample C3–W3 (containing 2.9/94.4/2.7 wt % of cellulose/bmimCl/water) in a capillary adopted the GA conformation after step 8 of low-temperature Treatment 2 (Table 4; spectra not shown), while the crystal form detected after step 5 was stressed monoclinic M, which transformed into monoclinic M0 ^39^ after step 9. It is reminded here that when Treatment 1 was applied to the same sample, the sample remained in an amorphous form throughout the whole course of Treatment 1 (Table 3).

## Discussion

### Treatment 1—The Impact of Cellulose Addition on the Structure of bmimCl

Addition of cellulose at a concentration of 1.0 wt % to neat bmimCl in a vial did not alter the resultant conformation of the butyl chain in the bmim cation when compared to the neat bmimCl sample in the same container, as reported earlier by Kotov et al.^39^—the AA rotational isomer was dominant in the binary cellulose/bmimCl mixture as well as in neat bmimCl (Table 3). Therefore, as has been reported previously,^43^ the ionic liquid solvent (bmimCl) was able to retain its inherent molecular arrangement and accommodate the macromolecular solute without having to alter the conformation of its butyl chain. Slower kinetics of ionic liquid crystallization were detected in the mixture with cellulose as opposed to the neat ionic liquid. While the equilibrium conformation was achieved already at step 2 in neat bmimCl in a vial, in the binary mixture cellulose/bmimCl the equilibrium was reached as late as step 5 of the low-temperature Treatment 1 (Table 3).

It has been shown that, if the chloride anion is not present in the vicinity of the butyl chain of bmimCl, the anion cannot form hydrogen bonds with the butyl chain and stabilize its GA conformation.^36^ Further, if the mechanism of cellulose dissolution proposed by Gross et al.^44^ is correct (the mechanism assumes that the anions in proximity to the cellulose microfibril surface disrupt the H-bonding network within the microfibril, thus enabling bmim cations to access the more hydrophobic faces of cellulose and intercalate between the flat cellulose sheets and ribbons), and if there is an interaction between the glucopyranose ring of cellulose and the aromatic ring of the bmim cation as proposed earlier,^11,18–25^ it could have been expected that a conformation other than GA is the preferred conformation of the bmim cation aliphatic chain when the cation is in direct contact with the cellulose macromolecule or, rather, the carbohydrate ring. The observation of the AA conformation in the vial sample of the 1 wt % cellulose/bmimCl mixture agrees with such a proposed mechanism and carbohydrate ring-aromatic ring interaction and with the conformation of the bmim cation that could be predicted on the basis of such a mechanism, however, it is not fully conclusive since conformation AA is also the preferred conformation in a vial of neat bmimCl, i.e., even when no cellulose is present.

In addition, all three capillary samples of the three cellulose mixtures (1 wt %, 3 wt % in this work, and 5 wt %;^25^ i.e., 1.1, 3.2, and 5.4 mol % AGU units, respectively) indicate the GA conformation as being dominant. Considering the fact that the GA conformation is dominant also in the capillary sample of neat bmimCl, we might only conclude that the forces that drove bmimCl to adopt this conformation in the neat ionic liquid in a capillary^39^ are also present in the capillary samples of bmimCl mixtures with cellulose. It is likely that, under suitable conditions, it is the AA conformation and orthorhombic crystal form that are the lower energy minimum compared to the GA conformation and monoclinic crystal form in neat bmimCl.^33^ In these energy minima, “docking” and packing of cation aromatic rings, van der Waals interactions between aliphatic butyl chains, and especially, hydrogen-bonding interactions between the chloride anion and aliphatic butyl chains are balanced and optimized. Obviously, such optimized structures might need to be altered when a (macromolecular) solute is present. However, the presence of extended cellulose macromolecules,^45,46^ even with possible carbohydrate ring–aromatic ring stacking^25,44^ and with disruption of hydrogen bonding between the chloride anion and bmim cation,^6^ while new interactions between cellulose hydroxyls and chloride anions are likely established, was not sufficient to establish the AA conformation throughout the bulk of the ionic liquid in the binary cellulose/bmimCl mixtures in capillaries. The propensity of the ionic liquids to maintain their inherent structure even upon solubilization of solutes was demonstrated once again.

### Treatment 1—The Impact of Cellulose Addition on the Kinetics of Crystallization

Opposite to the equilibrium structure of bmimCl, the kinetics of the ionic liquid crystallization was affected by the presence of cellulose solute—similar to the observation made for the vial sample above. The kinetics appeared to be slower in the mixture of ionic liquid with cellulose in capillary samples. While the equilibrium conformation in neat bmimCl in a capillary was achieved already at step 2, in the binary mixture cellulose/bmimCl the equilibrium conformation was reached at step 3 (1 wt % cellulose in bmimCl) and at step 4 (5 wt % cellulose in bmimCl; Kotov et al.^25^) of the low-temperature Treatment 1. The behavior of the 3 wt % cellulose/bmimCl sample was not fully conclusive (Table 3). These observations agree with the reports of Kotov et al.,^25^ who analyzed the relaxation times obtained from ssNMR spectroscopy and proved that the dynamics of all bmimCl carbons were slowed down upon the addition of cellulose. Such behavior was also described for other ionic liquids in mixtures with cellulose.^19,26^

The effect of cellulose on the kinetics of the structural transitions toward the equilibrium crystal structure, both in the vial and in the capillary, was most likely accompanied by a highly probable increase in the viscosity of the initial cellulose/bmimCl solutions compared to neat bmimCl. Since bmimCl is one of the most suitable solvents of cellulose, it likely dissolves this macromolecule so effectively that the individual macromolecules are free to adopt extended conformations.^45^ The surface area of such extended macromolecules is increased, and the attractive interactions between the polymer solute and ionic liquid solvent molecules are also increased, resulting in an increase of the solution viscosity^45^ thus affecting the molecular motion in the mixtures.

### Treatment 1—The Impact of Cellulose Addition on the Mixtures of bmimCl with Added Water: Concentration Effects

When a vial sample of the ternary cellulose/bmimCl/water (3.0/96.4/0.6 wt %) and the binary bmimCl/water (99.6/0.4 wt %) mixture, with similar added water concentrations by weight (0.6 and 0.4 wt %, respectively), are compared, their final conformations are the same—GA conformation was attained in both the ternary and in the binary mixtures in a vial, with the kinetics being faster for the binary mixture with added water.^39^ However, when the ternary mixture C3–W1 (3.0/96.4/0.6 wt % cellulose/bmimCl/water) reached equilibrium in a capillary, the final conformation was AA, and the crystal lattice was orthorhombic O, as observed by Kotov et al.^39^ but for the binary mixture A2 of a much higher added water concentration (97.1/2.9 wt % bmimCl/water). Such equilibrium was thus different from the GA conformation and monoclinic M0 lattice observed for the binary mixture A1 ^39^ that had an added water concentration (99.6/0.4 wt % bmimCl/water) rather close to the ternary mixture C3–W1 studied in the current work. One could presume that such differences in equilibrium structure were the combined effect of the presence of the extended macromolecule of cellulose and flow—especially if the mechanisms and interactions proposed previously^11,18–25,44^ that were summarized above, were taking place. First, however, it is necessary to assess the effect of the concentration of the solute (anhydroglucose units of cellulose and/or added water) in the ionic liquid on the adoption of the AA conformation and orthorhombic unit cell O in the ternary mixture, as opposed to the GA conformation and monoclinic M0 unit cell, when not weight but molar fractions are considered.

The molar fraction concentration of the ternary mixture C3–W1 is 3.1/91.5/5.4 mol % (anhydroglucose unit/bmimCl/water), which means that, on average, there are approximately 11 molecules of bmimCl per any one solute molecule (one anhydroglucose unit or one molecule of added water). In the relevant sample A1 of Kotov et al.^39^ (bmimCl/water 99.56/0.44 wt %, i.e., 95.9/4.1 mol %), there were as many as approximately 25 molecules of bmimCl per one added water solute molecule on average, and the sample adopted GA conformation and monoclinic unit cell M0. On the other hand, in sample A2 (97.1/2.9 wt %, i.e., 77.6/22.4 mol % bmimCl/water), there were as few as approximately five molecules of bmimCl per one added water solute molecule on average, and this sample adopted AA conformation and orthorhombic unit cell O. The number of bmimCl solvent molecules per one solute molecule in the current ternary mixture C3–W1 is 3.1/91.5/5.4 mol % (anhydroglucose unit/bmimCl/water), and this mixture thus falls between the number of bmimCl solvent molecules per one added water solute molecule in the two binary mixtures A1 and A2, while it is closer to mixture A2, which had the higher water content and conformation AA.

Further, it was discussed by Kotov et al.^39^ that there are likely several added water solute concentration regimes that determine the equilibrium structure of bmimCl. It would appear that, in terms of total (anhydroglucose unit and added water) solute concentration, the ternary mixture C3–W1 (3.1/91.5/5.4 mol % anhydroglucose unit/bmimCl/water) follows the behavior of the binary sample that has higher added water solute concentration, even though the total solute concentration in the ternary mixture is still lower (8.5 mol % as opposed to 22.4 mol %) than in A2 but higher (8.5 mol % as opposed to 4.1 mol %) than in A1.

Such behavior might simply indicate the approximate solute concentration intervals to which the different regimes are applicable. At the same time, it does not exclude the combined role of cellulose and hydrodynamic flow in imposing the stretched AA conformation upon the butyl chain of the bmim cation via the mechanisms and interactions proposed^11,18–25,39,44^ whereby the chloride anion interacts with cellulose hydroxyl groups and is prevented from forming a hydrogen bond with the butyl chain of the bmim cation, which, in turn, is prevented from adopting the conformation GA. In addition, the aromatic ring of the bmim cation probably stacks upon or docks onto the glucopyranose ring of cellulose.

The explanation of the crystallization behavior of mixture C3–W1, simply in terms of the solute concentration effect and in terms of various solute concentration regimes (regardless of what the solute is), would also hold true for the ternary mixture C3–W3 (2.5/76.2/21.3 mol % anhydroglucose unit/bmimCl/water). Mixture C3–W3 had a very similar content of added water to sample A2 studied by Kotov et al.^39^ when the concentration is expressed in weight percent: 2.7 wt % in C3–W3 and 2.9 wt % in A2. However, when subjected to Treatment 1, the ternary mixture C3–W3 did not crystallize and remained amorphous, while the binary mixture A2 crystallized on an orthorhombic O lattice and adopted the extended AA conformation. When solute concentration (either anhydroglucose unit plus added water concentration or added water-only concentration) in molar percent is considered, there are differences between these two mixtures. In binary A2, there are around five molecules of bmimCl per one added water solute molecule, while in ternary C3–W3, there are as few as about three molecules of bmimCl per one solute (anhydroglucose unit or added water) molecule. This makes this mixture very similar to the binary mixture A3 with about 3 bmimCl molecules per one added water molecule. Binary mixture A3 did not crystallize and remained amorphous throughout the whole low-temperature treatment (Kotov et al.^39^)—similar to the currently observed behavior of the ternary mixture C3–W3.

### Treatment 1—The Impact of Cellulose Addition on Mixtures of bmimCl with Added Water: The Effect of Partitioning and of Fluid Flow

It is possible that not only the total concentration of solutes in the ionic liquid is important, but the nature of the solute might also be significant. Since the cellulose macromolecule is likely to be the least mobile component of the ternary mixture, it is probable that, due to differing molecular properties (polarity, charge distribution, and others) of a water molecule and of a bmimCl molecule, the ionic liquid molecules and water molecules in both ternary mixtures, C3–W1 and C3–W3, are partitioning between those that are in contact with the cellulose macromolecule and those that are not. Such nanoscale partitioning is indicated by the phase separation of the water-rich phase observed in the ternary mixture C3–W1 at the end of Treatment 1. The formation of the water-rich phase in ternary mixtures of cellulose with bmimCl and added water undergoing low-temperature treatment implies there is at least one more phase that is depleted of water—most likely consisting predominantly of cellulose and ionic liquid (since cellulose is not soluble in water only), while the water-rich phase is most likely consisting predominantly of water with some ionic liquid—similar to the phase enriched in water observed by Kotov et al.^39^ for binary mixtures of bmimCl with added water. Further, such nanoscale partitioning would effectively divide the ionic liquid into (1) slower ionic liquid molecules that are localized along the extended cellulose macromolecules—the carbohydrate rings might possibly be interacting with the bmim cation, and the hydroxyl groups are interacting with chloride anions; the viscosity of this part of the ionic liquid would be increased compared to neat bmimCl—and into (2) faster ionic liquid molecules where bmimCl would be interacting mainly with water (viscosity would be decreased compared to neat bmimCl), while the water concentration in this faster ionic liquid would be higher than the overall added water concentration in the initial ternary mixture.This would place such partitioned bmimCl/water phase into the higher added water concentration regime (Kotov et al.^39^) for which the equilibrium conformation AA and orthorhombic unit cell O were detected (binary A2) or no crystallization took place (binary A3).

The abrupt changes in temperature from below zero to well above zero, which are taking place between the steps in both low-temperature treatments, are assumed to induce local fluctuations in the mixture density. Especially in the mixtures transferred into capillaries, such density fluctuations are thought to be coupled to capillarity—this results in hydrodynamic flow (as evidenced by the phase separation of the water-rich phase observed only in capillaries, which was partially discussed above). The nature of the flow—e.g., laminar vs turbulent—may vary depending on the precise balance of the density fluctuations and of capillarity phenomena. At the same time, the nature of the flow will affect the resultant equilibrium in the mixture studied: the hydrodynamic phenomena were shown to affect the crystallization of binary mixtures bmimCl/water^39^ and the solubility of polymers was proven to be significantly altered when the system flows.^47^ It is useful to remind that the flow pattern that takes place in a fluid is indicated by the Reynolds number *Re* (eq 1): if *Re* is lower than a certain value for the given geometry, then laminar flow is present; if it is above a certain value, then the flow is turbulent; and when *Re* is between these two values, the flow is transitional. *Re* increases with increasing density of the liquid, with increasing fluid velocity, as well as with increasing hydraulic diameter, but it decreases with increasing fluid viscosity (eq 1).

As already mentioned in the Introduction, it has been reported^4,31,32^ that the onset of turbulence during flow might be prevented by the addition of small amounts of polymer (this is called turbulent-drag reduction). At the micromechanical level of understanding the polymer–turbulence dynamics, drag reduction refers to the reduction of the Reynolds stress (the extra stress that is attributed to turbulent motion) as a consequence of polymer–turbulence interactions. Flexible polymers, especially, can alter fluid dynamics through an additional term in the momentum balance that is sometimes described as “polymer force.” The polymer force counters velocity fluctuations and counteracts vortices—in flexible polymers, such suppression of vortices is accompanied by the stretching of the polymer chain, while in rigid fibers, the alignment of fibers in intervortex regions was suggested.^48^ Suppression of vortex motion reduces the strength of velocity streaks in between, and this lowers the magnitude of Reynolds shear stress. Via polymer addition, such flow is achieved that has less fluctuations and more momentum retained in the mean flow. This is the drag reduction mechanism proposed for low-effect drag reduction; it might be different in high-effect drag reduction. There is no simple quantitative theory of polymer–turbulence dynamics. The suppression of the onset of turbulence can be understood in terms of the polymer damping the eddies, as mentioned above, while the effectiveness of the polymer in suppressing turbulence is, i.a., a function of the volume it occupies. In addition to averaging density fluctuations, the polymer added to the fluid can affect the local viscosity via another mechanism—the flow behavior is modified due to polymer interactions with neighboring solvent molecules.

Such a combined polymer effect on fluid flow very likely took place in the ternary mixture C3–W1, where the ionic liquid crystallized with the more extended conformation AA of the butyl chain than it did in the binary mixture A1 of Kotov et al.^39^—a mixture that was similar in terms of weight percent concentration of added water, but contained no polymer. Even though it is possible to argue that this effect on crystallization and conformation might be simply due to an overall increase of the concentration of solute when expressed as molar percent, regardless of what the solute is (see above), it is likely that such “molar concentration effect” might, in fact, be rather an effect of the fluid’s local density and viscosity. That is, the water-rich phase of the ternary mixtures (consisting predominantly of water and bmimCl—see the nanoscale partitioning described above) is considered to be the flowing fluid, while the water-depleted phase (predominantly cellulose with interacting bmimCl) is considered to be the nonflowing and more stationary polymer component. In such a system, the density of the fluid (predominantly water and bmimCl) is decreased compared to what the density would be based on the overall added water concentration in the initial ternary mixture. Such lower fluid density probably leads to a decrease in *Re* (eq 1), therefore leading to more laminar flow and to more extended conformations.

One could possibly also expect higher fluid velocity and lower viscosity as a consequence of decreased fluid density and faster motion. Higher fluid velocity and lower viscosity would subsequently increase *Re*. However, velocity and viscosity might not be changed significantly, since those ionic liquid molecules that are in the vicinity of cellulose macromolecules are presumed to be slowed down, as was indicated above by the slower kinetics of crystallization in binary mixtures of bmimCl with cellulose, both in vials and in capillaries. These slower ionic liquid molecules might prevent fully or partially reduce the acceleration of that part of ionic liquid molecules that are in the water-rich phase. Therefore, the overall fluid velocity and viscosity remained unchanged.

On the other hand, such polymer effect did not happen in the ternary mixture C3–W3 when compared to the binary mixture A2 of similar weight percent added water concentration (Kotov et al.^39^) during similar temperature treatment—Treatment 1. The opposite, while the binary mixture A2 crystallized (orthorhombic unit cell O, conformation AA), the ternary mixture C3–W3 remained amorphous. Such behavior would indicate that cellulose addition did not induce a reduction in *Re* via increased viscosity nor via averaging out the fluctuations. Possibly, since cellulose in C3–W3 occupies a smaller volume than in C3–W1, there might be less or no increase in viscosity, and the averaging of events by the polymer in the fluid might be less frequent. In addition, due to the suggested partitioning (ionic liquid in contact with cellulose vs ionic liquid in contact with water), the initial ternary mixture C3–W3 was likely transformed into domains containing cellulose macromolecules in interaction with bmimCl and domains that were predominantly solutions of water in bmimCl with an effective added water concentration higher than in the binary mixture A2. Therefore, any possible decrease in *Re* due to a decrease in fluid density (higher concentration of water due to partitioning) might be offset by a likely increase in fluid velocity and a decrease in viscosity—these changes would ultimately lead to an increase in *Re* and to turbulent flow that would prevent crystallization.

With hindsight, the fact that a binary mixture of ionic liquid with added water A1 in a capillary, in the work by Kotov et al.,^39^ adopted a monoclinic unit cell M0 and conformation GA that is not extended (unlike in a vial, where it crystallized on an orthorhombic lattice O with extended conformation AA), most likely indicates the type of flow pattern that was present in the binary mixture before equilibrium was reached. Even though this flow pattern did allow crystallization, it must have had some turbulent component—it might have been a transitional flow pattern. Following the same reasoning, temperature-induced density fluctuations in the capillary sample of binary mixture A2, with added water concentration higher than A1 and therefore with lower fluid density, and possibly higher velocity and lower viscosity, ultimately led to a more laminar pattern of flow in that work (Kotov et al.^39^), since the conformation of the butyl chains in the final crystallized state was the most extended one (AA). The “concentration effect” discussed above for ternary mixtures and in Kotov et al. in 2016 for binary mixtures^39^ might rather be an effect of water addition on the character of the flow and, in ternary mixtures, also an effect of water partitioning—i.e., an effect on the balance of the turbulent component and the laminar component. Such an effect is likely amplified when coupled to capillary flow, as was indicated by the fact that the structure adopted in a vial was different from the structure adopted in a capillary.

### Treatment 2—The Impact of Cellulose Addition on Mixtures of bmimCl with Added Water: The Effect of Fluid Flow

Low-temperature Treatment 2 is considered to be the milder of the two low-temperature treatments used in this study, since the magnitude of the temperature difference between the individual steps was smaller than in Treatment 1 (Table 2). Thus, since the temperature changes between steps of Treatment 2 are smaller, the density fluctuations in Treatment 2 are expected to be less pronounced and less likely to give rise to turbulent flow patterns, but the fluid velocity might be lower also. Such differences in flow patterns of Treatment 2, as compared to Treatment 1, have manifested themselves in the inability of ternary mixture C3–W1 to adopt one of the stable (not metastable) structures. Treatment 2 in C3–W1 only led to the metastable stressed monoclinic unit cell M and conformation GA, while in Treatment 1, the mixture was able to adopt the stable structure—orthorhombic unit cell O with conformation AA. This means that Treatment 2 did not provide enough momentum for transformation into the stable structure in the same way as was observed in Treatment 1. Such behavior would indicate that, in C3–W1, the transformation during Treatment 2 was too slow, i.e., the structure of the mixture was kinetically trapped and with reduced mass transport. The resultant conformation GA indicates that the flow was of a more turbulent character than in Treatment 1, upon the impact of which the more extended conformation AA was attained.

On the other hand, the ternary mixture C3–W3 was able to adopt one of the stable structures—monoclinic unit cell M0 and conformation GA—when subjected to the milder Treatment 2. Such behavior is very different from the behavior of C3–W3 displayed during Treatment 1, where this mixture remained amorphous due to the presumed turbulent character of the flow in Treatment 1. This implies that the flow patterns due to Treatment 2 had a less turbulent character than in Treatment 1, presumably due to lower fluid velocity, since the concentration fluctuations in the fluid (ionic liquid with water) were smaller and the flow present allowed it to adopt an ordered structure instead of the amorphous one.

Therefore, the ability of bmimCl to retain its inherent molecular arrangement, even upon solubilization of a solute that was confirmed in this work for binary mixtures of cellulose/bmimCl (see above), was overcome when the dynamic phenomena due to fluid flow became more prevalent in ternary mixtures cellulose/bmimCl/water, e.g., the conformation attained for the ternary mixture C3–W1 in a capillary after Treatment 1 was AA (Table 3), whereas the same mixture (C3–W1) in a capillary attained the conformation GA after Treatment 2 (Table 4), while the inherent conformation of neat bmimCl in a capillary is GA (Table 3).

### Generalized Comments

It might be suggested that the differences in the structural transitions and crystallization behavior documented here and in Kotov et al.^39^ for samples transferred into vials of 9 mm internal diameter and into capillaries of 2 mm internal diameter might not be caused by the differences in the size (in the diameter) of the respective containers, but might be due to differences in the glass of the containers. Such differences could be either differences in the detailed composition of the glass or in the structural details of the glass surface. However, the fact that the structural transitions were confirmed to be dependent on the details of the low-temperature treatment (i.e., Treatment 1 vs Treatment 2) that is applied to the capillary sample under investigation implies that fluid flow is affecting the crystallization of the ionic liquid, while fluid flow in a capillary is coupled to capillary phenomena. The capillary effect is thus relevant to the structural transitions: the different temperature treatments were coupled to capillary flow—a situation different from that of a vial sample—which, in turn, affected the process of crystallization.

The observations reported in this work indirectly indicate the presence of interactions between the cellulose macromolecule and its ionic liquid solvent. This is most clearly evidenced by the kinetics of crystallization of the binary mixtures of cellulose and bmimCl being slower than the crystallization of neat bmimCl, both in vials and in capillaries; in these binary systems, cellulose has an effect mainly on the kinetics of crystallization. Further, the presence of cellulose-bmimCl interactions is also indicated by the impact of cellulose on the structural transitions of bmimCl in ternary mixtures with added water in capillaries, when compared to the structural transitions of bmimCl in binary mixtures with added water in capillaries where no polymer was present. However, in the ternary mixtures, cellulose appears to affect the dynamics of the ionic liquid crystallization rather than its kinetics—such a dynamic effect is indicated, e.g., by the fact that ternary mixtures (C3–W1 and C3–W3) adopted different crystal structures when subjected to two different temperature treatments (cf. Tables 3 and 4).

It is not possible to specify in more detail what the actual interactions are in a way similar to Kotov et al.^25^, e.g., it is not possible to confirm directly the carbohydrate-aromatic stacking that was indicated by experiments in that work. However, current work does imply that the presence of cellulose may affect the resultant conformation of the butyl chain in the bmim cation and that cellulose can shift the equilibrium butyl chain conformation toward the more extended one, which, to the authors’ knowledge, has not been experimentally evidenced so far. However, such a shift in the equilibrium butyl chain conformation might not be a direct effect of cellulose macromolecule interactions—e.g., steric effects on the butyl chain or van der Waals forces—instead, cellulose macromolecules might be acting on the butyl chain indirectly via interacting with the imidazolium rings of bmim cations first, followed by water partitioning, thus enhancing the laminar component in the fluid flow. Even this interaction between the macromolecule of cellulose and the molecule of ionic liquid might be preceded by polymer–turbulence interaction first.

Such a suggested impact of intermolecular cellulose-ionic liquid interaction on the equilibrium conformation of the butyl chain would gain more importance if it were proven to be true that the length of the aliphatic chain in the ionic liquids is indeed optimized to be suitable for cellulose dissolution, as had been suggested previously.^23^ It could point toward the role aliphatic hydrophobicity may play in cellulose dissolution, since it would further confirm not only that both the cation and the anion of bmimCl are involved in the process, but it would also show that both the aromatic and the aliphatic moieties of the cation play a role in the dissolution.

## Conclusions

The work presented here assessed the impact of cellulose and added water on the kinetics of crystallization and the equilibrium structure of bmimCl when subjected to two different low-temperature treatments. The current study study was performed within the context of two preceding and closely related studies on similar systems carried out by Kotov et al.^25,39^ The ionic liquid bmimCl was shown to retain its ability to accommodate various—even macromolecular—solutes within its preexisting self-organized structure. However, the kinetics of the transformations toward the equilibrium structure were affected by the presence of cellulose macromolecules—it was slowed down. This effect was most likely caused by the number of interactions being maximized between the extended cellulose macromolecule and both the cation and the anion of bmimCl, as is typical for good polymer solvents. The behavior of mixtures where water was added to bmimCl in addition to cellulose (ternary mixtures) is strongly indicative of the effect of fluid flow on the conformation of the butyl chain of bmimCl and the crystal unit cell adopted. The final structures in ternary mixtures are most likely sensitive to the flow pattern being a laminar one, a transitional one, or turbulent. Such findings are highly relevant (1) to applications of both cellulose and ionic liquids, e.g., for the preparation of materials with precisely tailored nanostructures, (2) for both theoretical and practical studies of the impact of rigid polymers on fluid flow, and (3) to uncovering the mechanism(s) of cellulose dissolution.
